# Application of Metabolomics in Thyroid Cancer Research

**DOI:** 10.1155/2015/258763

**Published:** 2015-04-20

**Authors:** Anna Wojakowska, Mykola Chekan, Piotr Widlak, Monika Pietrowska

**Affiliations:** Maria Sklodowska-Curie Memorial Cancer Center and Institute of Oncology, Gliwice Branch, 44-101 Gliwice, Poland

## Abstract

Thyroid cancer is the most common endocrine malignancy with four major types distinguished on the basis of histopathological features: papillary, follicular, medullary, and anaplastic. Classification of thyroid cancer is the primary step in the assessment of prognosis and selection of the treatment. However, in some cases, cytological and histological patterns are inconclusive; hence, classification based on histopathology could be supported by molecular biomarkers, including markers identified with the use of high-throughput “omics” techniques. Beside genomics, transcriptomics, and proteomics, metabolomic approach emerges as the most downstream attitude reflecting phenotypic changes and alterations in pathophysiological states of biological systems. Metabolomics using mass spectrometry and magnetic resonance spectroscopy techniques allows qualitative and quantitative profiling of small molecules present in biological systems. This approach can be applied to reveal metabolic differences between different types of thyroid cancer and to identify new potential candidates for molecular biomarkers. In this review, we consider current results concerning application of metabolomics in the field of thyroid cancer research. Recent studies show that metabolomics can provide significant information about the discrimination between different types of thyroid lesions. In the near future, one could expect a further progress in thyroid cancer metabolomics leading to development of molecular markers and improvement of the tumor types classification and diagnosis.

## 1. Introduction 

Thyroid carcinoma is a common endocrine malignancy in the head and neck region. There are several histological types of thyroid cancer, including papillary thyroid carcinoma (PTC), follicular thyroid carcinoma (FTC), medullary thyroid carcinoma (MTC), and anaplastic thyroid carcinoma (ATC). Determining the type of thyroid cancer is crucial for the assessment of prognosis and treatment selection. Most patients with thyroid carcinoma are initially diagnosed based on the result of fine needle aspiration cytology. Refined diagnosis and classification/staging of this cancer are available after surgery based on histopathological examination of the resected tissue. Unfortunately, in some cases, proper classification could be problematic when histopathological patterns are inconclusive [1]. Therefore, classical histopathological approach in the diagnosis of thyroid cancer could be potentially supported by molecular biomarkers. Nowadays, a number of molecular tests to confirm the diagnosis of thyroid nodules have been proposed, which included panel of somatic mutations (e.g., RET-PTC, RAS) and immunocytochemistry tests (e.g., BRAF V600E IHC) [2–4]. This approach, although not implemented widely in clinical practice yet, could change the attitude towards classification of thyroid cancers in near future [5]. Further application studies and clinical trials hold great promise for molecular support of classical thyroid cancers diagnosis. Such applications are likely to become a component of the standard diagnostic approach for patients with thyroid cancer. Therefore, there is an urgent need to determine the most cost-effective protocols to utilize these molecular-based diagnostic tools.

Metabolomics is one of the high-throughput “omics” techniques, which beside genomics, transcriptomics, and proteomics play an important role in systems biology. Metabolome is the final downstream product of gene expression and therefore reflects changes in the transcriptome (mRNA) and the proteome (proteins). Additionally, metabolomics reflects phenotypic changes and alterations in pathophysiological states of biological systems and therefore represents the most “downstream” level of molecular life of a cell. Metabolomics targets different classes of low molecular weight (MW < 1500 Da) metabolites. In contrast to plants, human metabolomes are relatively well known, defined, and catalogued. The estimated number of components of human metabolomes ranges from thousands to tens of thousands, depending on type of a cell or tissue [6]. Changes in metabolome composition reflect alterations in enzymes concentration, cellular regulation, control of signalling pathways, genetic variations, and catabolic and anabolic reactions. Therefore, metabolome most directly reflects the phenotype, physiology, and molecular state of an organism [7]. The main drawback of metabolomic studies, reflecting chemical variability of this cellular component, is the fact that there is no single analytical method allowing simultaneous measurement of such broad spectrum of bioactive compounds. Hence, different combinations of liquid (LC) and gas (GC) chromatography coupled with mass spectrometry (MS) or nuclear magnetic resonance (NMR) are the most accepted and widely used analytical approaches in this field [8].

Metabolomics provides valuable information about metabolism of malignant cells and has a great potential in cancer research as well as in identification of novel diagnostic and prognostic markers [9]. Several studies proved that metabolomics approaches allowed for classification of different types of malignancies and identification of potential biomarkers in the case of brain [10], breast [11], kidney [12], and prostate [13] cancers. Although studies regarding metabolome of thyroid cancer are not very common, there are several recent works showing that metabolomics approach could help to discriminate different types of thyroid lesions and provide significant information about their progression. Here, we aim to review recent progress in the field of thyroid metabolomics and discuss its contribution to understanding thyroid tumorigenesis and potential refinement of molecular classification of thyroid cancers.

## 2. Dilemmas in Thyroid Cancers Diagnosis 

Palpable thyroid nodules occur in 4–7% of the population; however, lesions found incidentally during ultrasonographic examination suggest a prevalence of 19–67%. The classification of such nodules includes numerous entities, both nonneoplastic and neoplastic, benign and malignant. Although thyroid tumors occur in roughly 5–10% of palpable nodules, they represent the most common endocrine malignancy and pose a significant challenge to pathologists, surgeons, and oncologists [14]. Fine-needle aspiration (FNA) cytology is a simple, rapid, inexpensive, and minimally invasive procedure which plays an important role in decision-making regarding clinical management of patients with thyroid nodules. Nowadays, the Bethesda system for reporting thyroid cytology [15] is generally accepted. It has six diagnostic categories, which correlate with risk of malignancy, and provides clear management guidelines to clinicians to go for follow-up FNA or surgery [16]. In general, many thyroid cancers can be diagnosed with certainty using FNA, yet the method has some limitations. For example, the nuclear and architectural changes of some PTCs are subtle and focal. This is particularly true of the follicular variant of PTC, which can be difficult to distinguish from a benign follicular nodule. Other PTCs may be incompletely sampled and yield only a small number of abnormal cells. Some rare benign lesions like hyalinizing trabecular tumors are frequently misdiagnosed by FNA cytology as PTC [17, 18]. Histological distinguishing of follicular carcinoma from follicular adenoma is based on the presence of capsular or/and vascular invasion, whose features could not be analyzed in cytological material. Moreover, the cytological features alone cannot reliably separate the detected thyroid tumors into benign or malignant; hence, patients with lesions are advised to undergo a diagnostic surgery. In fact, less than 30% of such lesions were diagnosed after surgery as malignant [19]. Therefore, several molecular tests for adjustment of cytological diagnosis of thyroid nodules have been proposed, which included panel of somatic mutations (e.g., BRAF and NRAS mutation and/or RET/PTC translocation) and immunocytochemistry tests based on gene expression signatures. BRAF gene mutation has been detected in 30% to 80% of PTC [20]. BRAFV600E is very specific for PTC, yet the failure to detect this mutation does not rule out PTC. Nevertheless, analysis of BRAF mutations improved diagnostic yield in cases of indeterminate cytology [21]. Another approach proposed recently to improve diagnostics based on FNA material included commercially available tests: Afirma Gene Expression Classifier for Thyroid FNA Analysis and miRInform Thyroid Test by Asuragen [22, 23].

When concerning postoperative material, a reliable histopathological diagnosis can be reached by an experienced pathologist by sole morphologic assessment for most cases of thyroid tumors. However, several types of morphologic features (e.g., the presence of nuclear atypia) are not sufficient for reliable diagnosis of malignancy. For example, in Hashimoto's thyroiditis and dyshormonogenesis, isolated cells with pleomorphic nuclei are very common. Furthermore, in the case of tumors exhibiting unusual morphologic patterns or in order to confirm the diagnosis of medullary carcinoma, additional molecular tests (e.g., immunohistochemistry) are required. Nevertheless, the currently used molecular tests have only limited application in routine diagnosis of thyroid neoplasia. Although several IHC tests (e.g., for cytokeratin-19 and galectin-3) have been proposed for distinguishing malignant from benign thyroid lesions, they frequently show high level of false-positive and false-negative results [24]. Also molecular markers suggested for differential diagnosis of PTC versus benign thyroid lesions or other thyroid tumors have been widely tested in clinical practice. In fact, certain normal thyroid follicles, nonneoplastic thyroid lesions (in particular thyroiditis), and benign thyroid tumors can exhibit focal or extensive staining for many of putative “thyroid cancer markers.” For instance, 31–55% of adenomatous hyperplasia could be positive for cytokeratin-19 or galectin-3. However, even application of a panel of markers for IHC studies (e.g., Ck-19, Gal-3, and HBME-1) did not improve significantly the diagnostic performance of the test [25]. The apparent role of interactions between the tumor and its niche in the tumor-thyroid interface represents additional diagnostic challenge. Recently, promising results have been reported for the usage of CD56 for differentiation of benign lesions and PTC [26–28]. Additionally, in the case of a follicular patterned neoplasm lacking the cytoarchitectural features of papillary carcinoma, the only feature that distinguishes carcinoma from adenoma is the presence of unequivocal vascular and/or capsular invasion in the former [29].

In conclusion, histopathological evaluation remains the gold standard in the distinction between different types of thyroid cancer, for example, between follicular carcinoma and follicular adenoma. Various molecular tests have been tested beside histological analysis, including immunohistochemical assessment of proteins and genetic tests for gene mutations, yet none of them proved its actual clinical applicability. Therefore, there is a constant need for development of unambiguous molecular markers which would bring a real improvement in diagnosis and classification of thyroid cancers.

## 3. “*Omics*” Approaches in Thyroid Cancer Research

The term “omics” defines high-throughput approaches to complex molecular composition of tissues enabling simultaneous analysis of thousands of genes/proteins. The main assumption of systems biology is integration of different “omics” datasets in order to obtain a broad perspective on the complex processes occurring in a living organism. For this purpose, platforms integrating genomics, transcriptomics, proteomics, and metabolomics data are constructed, enabling better understanding of mechanisms involved in natural history of cancer. Therefore, there is a generally expressed expectation that such approach would also deliver new biomarkers to be applied in clinical practice [30].

Microarray and next-generation-sequencing approaches to the analysis of gene expression as well as mass spectrometry techniques used for the analysis of proteins and/or peptides have delivered valuable information on various types of human malignancies. The best example of how the achievements of the genomics era could alter the clinicopathologic paradigm in classification of cancer types and affect the decision making process in selection of a treatment is breast cancer [31–34]. Similar “omics” approaches have been tested during the last decade for many other malignancies, including thyroid cancer. The first global microarray-based gene expression profile of thyroid cancer was reported in 2001 [35], when the gene signature characteristic for papillary thyroid cancer was identified. Another microarray-based study allowed for identification of the gene expression signatures associated with mutations in BRAF, RAS, and RET/PTC genes, as well as distinguishing the classic PTC from the tall cell and follicular variants [36]. Comparative analysis of the expression profiles in PTC and FTC, the two most common forms of thyroid carcinoma, enabled identification of the gene signatures characteristic for these cancers; the differentiating signature includes five genes (CITED1, CAV1, CAV2, IGFBP6, and CLDN10) [37]. Since then many other works have been published describing the differences between thyroid cancer and normal thyroid tissue, as well as differences between types of thyroid neoplasia. These works revealed the significance of hundreds of genes, including the key genes involved in thyroid hormone biosynthesis [38, 39]. An important issue in the diagnostics of thyroid cancer is differentiation between follicular adenoma, follicular carcinoma, and the follicular variant of papillary carcinoma. Currently, several biomarkers have proved their applicability in solving this problem, including LGALS3, hemoglobin, epsilon 1 (HBE1), keratin 19 (CK-19), and TPO (thyroid peroxidase) [40].

Another powerful and promising approach in the search of cancer biomarkers is proteomics based on mass spectrometry tools [41–45]. Proteins can be extracted, identified, and quantified from different cell and tissue sources. It should be stressed that proteomics studies of thyroid represent a real challenge due to high heterogeneity observed in this tissue and very broad range between the most abundant (e.g., thyroglobulin) and the least abundant proteins. Important disease-related proteins are likely to be discovered in the subpicomolar range, which means that thyroid proteome profiling methods must cover the dynamic range substantially wider than 10^10^ [46]. Nevertheless, several papers addressing the differences between thyroid lesions have already been published. The comparative analysis of PTC specimens matched with the normal thyroid tissue from the same patients and benign follicular adenomas allowed identification of three proteins, namely, S100A6 (an isoform of S100 protein), peroxiredoxin 2, and heat shock protein 70 (HSP70), whose expression levels were markedly higher in PTC tissue. Furthermore, the study confirmed overexpression of several PTC markers identified earlier by mRNA study (e.g., galectin-3, cytokeratin-19, and cathepsin-B) [47]. Another study compared proteome profiles between thyroid follicular adenomas and follicular carcinomas and revealed statistically significant difference in abundance of 43 proteins detected in thyroid tissue [48].

Genomics and proteomics studies have a significant impact on general understanding of cancer-related processes and have delivered several promising candidates for cancer biomarkers. However, some limitations in the prognostic and prediction power of gene/protein expression data have been recognized in recent years. The challenge is that correlation between the established mRNA and protein expression levels and their real influence on phenotype of cancer has appeared elusive in many cases. Therefore, other approaches directly addressing phenotypic features of a cancer tissue, represented by metabolomics, have a large potential in cancer studies.

## 4. Cancer Metabolism

**Figure 1:**
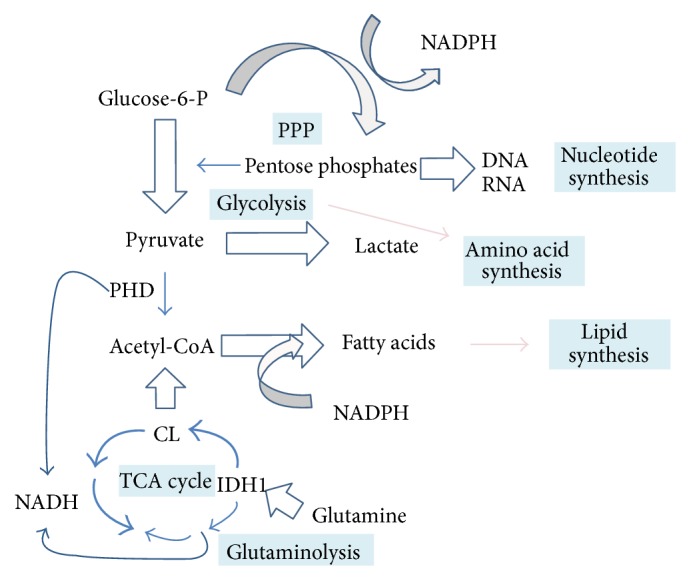
A simplified scheme of metabolic pathways in cancer cells (modified from Heiden et al. [51] and Denkert et al. [11]). Thickness of arrows indicates relative intensity of fluxes. PDH: pyruvate dehydrogenase; CL: citrate lyase; IDH1: isocitrate dehydrogenase 1.

Cancer progression is a complex process which involves proliferation, hypoxia, angiogenesis, apoptosis, metastasis, inflammation, and increased tolerance to reactive oxygen species [49]. These tumor associated processes significantly affect the primary metabolic pathways; hence, cancer cells are characterized by altered metabolism in comparison with the normal differentiated cells [50], whose pathways are depicted schematically in Figure 1.

The major difference between cancerous and normal cells concerns the pathways involved in production of energy. In healthy tissues, glucose is used for the production of NADH and ATP during the tricarboxylic acid cycle (also known as the Krebs cycle or the citric acid cycle) and oxidative phosphorylation. In marked contrast, most cancer cells use aerobic glycolysis, known as the Warburg effect, to produce both energy and “building blocks” (amino acids, nucleotides, and fatty acids) needed for extensive growth and proliferation [51]. Otto Warburg was the first to observe that tumor cells take up large amounts of glucose which is converted to lactic acid [52]. Since then numerous studies have shown the increased level of lactate and other glycolytic products in cancer tissues [53–55], which is a phenomenon that can be used in clinical practice for detection of tumors.

Cancer metabolome is also characterized by elevated amounts of fatty acids and lipids, which are essential for cell membrane building. Fatty acids are synthesized* de novo* during cancer progression [56–58] which requires NADPH and acetyl-CoA. In cancer cells, NADPH is produced via increased glutaminolysis and the pentose phosphate pathway (PPP) [59]. PPP, which is upregulated by glycolysis, provides pentose phosphates which are essential also in the synthesis of nucleotides. Moreover, increased glutaminolysis provides acetyl-CoA in the reverse reaction of citrate synthase [60]. In addition to NADPH and acetyl-CoA, choline is the next essential substrate required for lipids biosynthesis, and accumulation of this compound in cancer cells was observed in different studies [61]. Choline-containing compounds, including phosphocholine, phosphatidylcholine, and glycerophosphocholine, are the key components of a cell membrane. Phospholipids are the major constituents of cell membranes, determining their shape and fluidity [62]. Thus, alterations in membrane phospholipids may influence key aspects of cancer phenotype like invasiveness and metastatic potential [63]. Changes in the levels of lipids and their derivatives were observed in patients with different type of malignancies, including breast [64], prostate [65], brain [66], and thyroid [67] cancers. Saturated and unsaturated lipids forming a cell membrane could move through the cytosol as mobile droplets and accumulate in cytosolic vesicles. It was reported that such mobile lipids were associated with tumor progression [68]. Currently, it becomes widely accepted that lipids associated with proliferation and inflammation have apparent prognostic value in cancer diagnostics [69, 70].

In addition to lipids, there is a large group of small metabolites, including amino acids, nucleotides, sugars, and organic acids, important for cancer development. As a consequence of the Warburg effect, the decreased level of glucose and simultaneously increased levels of lactic acid and alanine are observed, especially during hypoxia and ischemia [71–73]. Levels of taurine and myo-inositol, other small molecules involved in osmoregulation, are also affected in cancer, which was reported in thyroid, prostate, colon, breast, and ovarian cancers [11, 55]. Although elevated levels of phospholipids and products of glycolysis characterize cancer cell in general, it should be emphasised that specific levels of various metabolites, including glycine, alanine, lactate, citrate, nucleotides, and lipids, may depend on the type of a cancer [12]. Consequently, metabolomics approach allows distinguishing not only between the normal and cancer tissue but also between different types and stages of malignancy.

There are several types of cancer whose metabolome is relatively well-characterized. Breast cancer is generally characterized by elevated level of total choline-containing compounds (tCho), low glycerophosphocholine, and low glucose, when compared to healthy tissues and benign tumors [64, 74–77]. Furthermore, specific differences in features of metabolome were observed between different histotypes of breast cancer [13]. Another example of well-described malignancies is brain tumors, whose different histological types showed distinct metabolic profiles reflecting the levels of alanine, threonine, creatine, glutamate, and phosphocholine [78–80]. Also the metabolome of prostate cancer was characterized, where high levels of tCho and phosphocholine, along with increased amounts of alanine and lactate, were observed [81]. Therefore, metabolomics has an apparent potential in the studies focused on the discovery of cancer biomarkers. Current research efforts are focused on the use of metabolomics screening in preclinical and clinical studies to improve diagnosis and support therapy. However, there is still a significant need to establish the rigorous and effective analytical protocols which could be widely accepted and find their appropriate place in clinical trials.

## 5. Methods of Metabolomics

**Figure 2:**
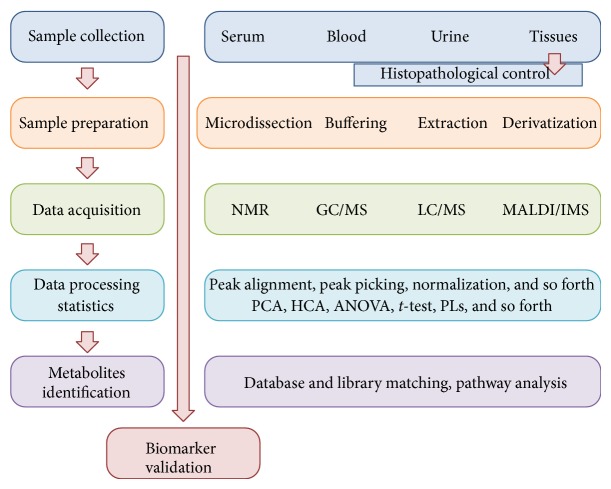
The general workflow of metabolomics analysis in cancer research.

The number of metabolites present in a human organism is currently estimated as approximately 17,000 (according to The Human Metabolome Database-HMDB version 3.6), yet this number is still expanding; hence, the exact figure remains unknown. Due to extremely diverse physicochemical properties of different metabolites and highly dynamic changes in the composition of the metabolomes, there is no single analytical method allowing examination of the entire metabolome. Analytical approaches implemented in this field vary in their specificity and sensitivity.* Metabolomic fingerprinting* is the least precise approach which enables rapid monitoring of the composition of low molecular weight compounds, without the need for detailed identification. The most commonly used method is* metabolite profiling*, allowing qualitative and quantitative analysis of a given group of metabolites.* Targeted analysis* enables the most detailed study of a selected class of compounds [10, 82]. Regardless of the chosen analytical approach, there are several general steps in the metabolomics studies, including sample collection and preparation, data acquisition and processing, biostatistical analysis, and data interpretation, which have been schematically depicted in Figure 2. In order to obtain reproducible results that could be compared between laboratories, strict compliance with the standardized procedures of metabolomic analysis is required. For this purpose, Metabolomics Standard Initiative (MSI) (http://www.msi-workgroups.sourceforge.net/) published standard reporting requirements for each step of metabolomics experiments [83–87].

The first and also the most critical steps in metabolomics study are sample collection, storage, and preparation for instrumental analysis. Blood (and its derivatives like serum) and urine are the most commonly used specimens for biomarker studies because of easy repetitive access and high yield. Moreover, saliva, breath condensate, bronchial washes, pancreatic juices, prostatic secretions, faeces, and other types of physiological liquids or surrogate tissues can be also used for metabolomics studies [12]. For different biofluids, the standard sample volume required for most types of analyses is within the range of 0.1 to 0.5 mL. Most obviously, tissue specimens are also widely examined in metabolomics studies using mass spectrometry (MS) [88], imaging mass spectrometry (IMS) [89], and nuclear magnetic resonance (NMR) spectroscopy [90] techniques. However, tissue samples are usually characterized by large heterogeneity and therefore require more complex preparation procedures before analysis [91], including the use of laser-capture microdissection (LCM) techniques [92]. The collected material can be fresh-frozen and stored in the temperature below −80°C, which is a gold standard in the analysis of metabolites. However, tissue specimens fixed in formalin and stored as formalin-fixed paraffin-embedded (FFPE) samples (normally stored in room temperature) are the most accessible source of clinical material for molecular studies. This type of tissue material is also suitable for certain metabolomic analyses when properly collected and stored [93]. Each type of specimen is characterized by different features, for example, volatility, extraction efficiency, and storage requirement. Nevertheless, due to high sensibility of metabolites to exogenous environment, maintaining low temperature and selection of the appropriate method of extraction are essential in most of the cases.

Metabolites present in biological samples differ in terms of molecular weight, thermostability, volatility, and polarity. Therefore, it is impossible to isolate all of them simultaneously, using the same method of purification. Consequently, the choice of the sample preparation method depends on the character of the studied compound class and the type of the analytical technique [94]. For isolation of a relatively large group of metabolites, organic solvents of different polarity (i.e., methanol, acetonitrile, chloroform, or hexane) should be used for analyte extraction. Additionally, separation techniques based on gas chromatography require further derivatization of analytes, which enhances their volatility [95]. The techniques based on NMR require less complicated procedures for sample processing, yet these analytical methods are less sensitive when compared to MS [96].

The most commonly applied analytical methods in cancer metabolomics studies are liquid or gas chromatography coupled with mass spectrometry (LC/GC-MS) and nuclear magnetic resonance (NMR) spectroscopy [91]. These two powerful techniques have both their advantages and limitations. MS is a highly sensitive technique which allows identification and quantification of multiple metabolites, even at very low concentrations, based on the mass to charge ratio of the analyte ions generated in the spectrometer [97]. High-resolution MS^n^ experiments with sequential fragmentation of the analyte ions permit obtaining structural information about the studied compounds [98], yet not all metabolites can be ionized using either positive or negative ionization mode. Chromatographic techniques coupled with MS allow separation of complex mixture of analysed compounds, which is a typical problem in the case of biological materials. GC/MS approach requires more time-consuming sample processing, yet it is a reproducible method allowing for access to large databases for automatic identification of metabolites [99]. NMR spectroscopy also enables identification of small biomolecules based on the resonance spectra of the atomic nuclei ^1^H, ^13^C, and ^31^P, which are commonly present in metabolites [96]. Methods based on NMR are generally less sensitive in comparison to MS techniques; however, they can be used in analyses of liquid and solid samples with minimal preparation stage. High-resolution magic angel spinning (HR-MAS) NMR spectroscopy is widely tested for metabolite-based cancer tissue classification [55, 100]. More recently, direct analysis of spatial distribution of metabolites in a tissue is an emerging approach in metabolomics. Spatial distribution of metabolic markers can be analyzed both* in vitro* and* in vivo* using localized magnetic resonance spectroscopy imaging (MRSI) [101]. Another method for* in vitro* differentiation of tissue regions based on their molecular content is imaging mass spectrometry (IMS). IMS allows for high-resolution determination of spatial distribution of metabolites and lipids within the tissue, which was utilized in several studies oriented on cancer metabolome [102–104]. The combination of different analytical methods, including MS, NMR, and imaging techniques, apparently provides the most powerful approach to metabolomics studies in current and future applications [13].

The last stage of a metabolomics study is data analysis and interpretation, including several different computation and bioinformatics steps. The collected MS or NMR spectral data requires multistep processing and normalization for further identification of components and their quantification [105, 106]. Subsequently, the registered molecules are identified by annotation at metabolomics databases, such as Human Metabolome Database (HMDB) [107], METLIN [108], Golm database [109], MassBank [110], or LIPID MAPS [111]. Next, multivariate statistics, including supervised and/or unsupervised methods, is required for pattern-recognition and identification of multicomponent signatures or classifiers characteristic for different biological states (e.g., tumor versus normal tissue). Supervised methods (e.g., PLS-DA, OPLS-DA) use the information of class membership to classify a given dataset. Unsupervised approaches (e.g., PCA, HCA) have been applied to investigate the innate variation in dataset [112]. Nevertheless, biomarker candidates obtained in existing studies apparently require further testing and validation using independent material and (most preferably) cross-validation studies involving interlaboratory cooperation [113, 114].

## 6. Metabolomics in the Studies on Thyroid Cancer 

There are numerous works proving successful application of genomics, transcriptomics, and proteomics in the field of thyroid cancer research [2, 46, 115]. In contrast, metabolomic studies concerning this malignancy are currently limited to relatively few papers. However, the metabolomics approach implemented in identification of biomarkers for diagnosis and classification of thyroid tumors has been dynamically expanding in recent years [55, 58, 67, 116–118]. The state-of-the-art technologies including MALDI-IMS (Matrix Assisted Laser Desorption Ionization-Imaging Mass Spectrometry) and HR-MAS NMR (High-Resolution Magic Angle Spinning Nuclear Magnetic Resonance) have been used for the identification of a large group of metabolites present in thyroid tissues, which are potential diagnostic biomarkers [58, 116]. In general, significant differences were observed between the metabolomes of normal thyroid tissues and neoplastic lesions, as well as between benign and malignant nodules.  Table 1 summarizes findings of key papers published in the field. Major groups of metabolites, whose different abundance in thyroid tissue and/or blood was observed between different types of thyroid lesions, are listed there.

### 6.1. NMR-Based Analyses of Metabolome Discriminate Different Thyroid Lesions

Discrimination of different thyroid lesions, such as nonneoplastic nodules, follicular adenoma, and malignant tumors, based on metabolite profiles constitutes a technically challenging but clinically relevant problem. The first studies demonstrating the potential of ^1^H NMR to differentiate between normal and malignant thyroid metabolites were conducted during the last decade of 20th century [119–122]. Shortly afterwards, the potential of NMR techniques in the analysis of lipid component of thyroid cancer was reported [123, 124]. More recently, HR MAS ^1^HMR and MRI methods have been applied to distinguish between benign and malignant thyroid nodules [125, 126]. In fact, NMR metabolomic data processed with multivariate statistical approaches allowed separation between different types of thyroid lesions and several multicomponent metabolic signatures/classifiers were proposed. Miccoli et al. [55] took advantage of HR MAS ^1^HMR and noted elevated levels of several amino acids (mainly phenylalanine and taurine) and lactate combined with decreased levels of choline (and its derivatives) and scyllo-/myo-inositol in malignant lesions (PTC, FVPTC, and FTC) in comparison to the benign ones, yet significant differences between the different cancer histotypes were not detected. The observed changes in the levels of lactates and inositols were in agreement with the changes generally observed in many types of tumors. However, the decrease in the content of choline and its derivatives was not generally observed in cancer tissue [57, 127, 128], also in other works on thyroid cancer [58, 67, 118, 129, 130]. Deja et al. [116] implemented ^1^H NMR technique in analysis of aqueous tissue extracts of healthy thyroid tissue, nonneoplastic nodules, follicular adenomas, and malignant cancer. The authors subjected acquired NMR data to multivariate analysis including both unsupervised method (PCA) and supervised modelling (OPLS-DA) and identified metabolites characteristic for different types of thyroid lesions. Potential biomarkers common to all thyroid lesions included alanine, methionine, glutamate, glycine, tyrosine, phenylalanine, hypoxanthine, acetone, and lactate. The reduced levels of scyllo- and myo-inositol, which act as osmoregulators, were found to be specific for thyroid cancers. Moreover, a decrease of lipids content in malignant lesions was detected when compared to the healthy tissue. The increased level of lactate was found not only in cancer tissue but also in other thyroid lesions. In general, the observed features of the cancer metabolome reflected dysregulation of the tricarboxylic acid cycle, ammonia recycling, amino acids metabolism, and osmotic regulation. Based on the specific metabolite profile of follicular adenomas and significant differences between healthy control, nonneoplastic nodules, and thyroid cancer, the authors classified this lesion as an intermediate step in thyroid cancer progression. This observation gives a promise of new potential metabolomic biomarkers of different thyroid cancer stages.

### 6.2. IMS Reveals Specific Features of Thyroid Cancer Lipidome

Imaging mass spectrometry (IMS) has already been used for simultaneous detection and spatial localization of lipids in differentiated thyroid cancer tissues. Ishikawa et al. [67] focused on distribution of phospholipids in papillary carcinoma in comparison to normal thyroid tissue. They identified phosphatidylcholines PC (16:0/18:1), PC (16:0/18:2), and sphingomyelin SM (18:0/16:1), whose levels in PTC were significantly higher than in nontumor tissue. More recently, Guo et al. [58] presented the results of their work where molecular tissue imaging using MALDI-FTICR-IMS was combined with serum lipidome profiling. The study was focused on phosphatidylcholines (PC), phosphatidic acids (PA), and sphingomyelins (SM) in three types of tissues: normal thyroid, benign tumors (thyroid adenoma and multinodular goiter), and malignant tumors (papillary and follicular thyroid carcinoma). Based on IMS analysis performed on 36 tissue samples and LC/MS profiling of almost 300 sera from three different groups of patients (healthy, with malignant thyroid tumor, or with benign thyroid tumor), the authors identified ten differentiating lipid species: phosphatidylcholines PC (34:1), PC (36:1), PC (38:6), phosphatidic acids PA (36:2), PA (36:3), PA (38:3), PA (38:4), PA (38:5), PA (40:5), and sphingomyelin SM (34:1). These potential biomarkers (or their subpanels; see Table 1) detected in serum showed potential diagnostic power to differentiate between cancer patients and healthy individuals as well as between patients with malignant and benign thyroid lesions. Moreover, the authors validated the previous findings [56, 57] that* de novo* synthesis of fatty acids was associated with tumorigenesis. For this purpose, expression of key enzymes, SCD1 and FASN, involved in* de novo* fatty acids synthesis [131, 132] was correlated with the levels of monosaturated PC in malignant, benign, and normal thyroid tissues. Immunohistochemical detection of SCD1 and FASN confirmed their association with high level of monosaturated PC in thyroid cancer tissues.

The impact of lipid metabolism on thyroid tumorigenesis has also been shown by Yao et al. [118]. The authors conducted serum metabolic profiling of PTC, nodular goiter cases, and healthy control using LC/MS technique. Significant changes in the levels of amino acids, free fatty acids (FFA), and phospholipids were observed between different groups of donors. Moreover, the most significant differences between benign and malignant nodules were observed in lipid metabolism. The deregulation of lipid metabolism in a group of patients with PTC was mirrored by an increased level of sphingosine, FFA, 3-hydroxybutyric acid, and carnitine. Particularly, the level of 3-hydroxybutyric acid, which is intermediate product of fatty acid metabolism, was significantly higher in patients with PTC than in benign tumors and healthy control cases.

## 7. Conclusions

Metabolomics has an apparent potential to expand our knowledge on molecular factors involved in thyroid cancer. Recent works have indeed shown significant differences in metabolomes of normal and neoplastic thyroid tissues, as well as between various stages of neoplasia. However, the example of breast cancer studies clearly indicates the need for combining the metabolomics information with other systems biology datasets to provide the holistic view of the processes ongoing in this endocrine malignancy. Such metabolomics network has already been created for thyroid hormone secretion pathway, which helped to identify the potential drug targets essential in treatment of thyroid disorders such as hypo- or hyperthyroidism [133, 134]. Along with the technological development of analytical tools (first of all MS- and NMR-based spectral techniques), metabolomics becomes an emerging research approach also in the field of thyroid cancer. However, despite the promising value of initial studies, there is a need for rigorous standardization of analytical methods and validation of preliminary results using large independent datasets. Moreover, there is an obvious necessity for integration of metabolomic data to genomics, transcriptomics, and proteomics results to bring better insights into the cellular mechanisms of thyroid cancer progression. Nevertheless, one should expect that metabolomics studies may deliver in the next years the basic knowledge not only on cancer-related processes but also on novel biomarkers to be implemented in diagnosis and classification of thyroid cancer.

## Figures and Tables

**Table 1:** A summary of metabolomic studies performed on thyroid cancer. This table contains only references to the studies for which the number of samples was representative and the results were statistically significant.

Reference	Source material	Thyroid sample type	Analytical method	Main results
Deja et al., 2013 [116]	Thyroid tissue	Thyroid carcinoma; follicular adenoma; nonneoplastic nodules (hyperplastic, colloid, and cystic nodules); healthy control	^1^H NMR	Discrimination of thyroid lesions (TC, FA, and NN) from healthy control (HC) as well as between different thyroid lesions was reported Potential biomarkers/discriminators: (i) TC, FA, and NN > HC: lactate, alanine, methionine, glutamine, glycine, tyrosine, phenylalanine, hypoxantine, and taurine; (ii) TC, FA, and NN < HC: acetone, myo- and scyllo-inositol, and lipids; (iii) FA > NN: branched chain amino acids (BCAA); (iv) FA > NN: citrate and N-acetylated compounds; (v) FA > TC: myo- and scyllo-inositol; (vi) FA < TC: lactate, methionine

Miccoli et al., 2012 [55]; Torregrossa et al., 2012 [117]	Thyroid tissue	Thyroid carcinoma (PTC, FVPTC, FTC); follicular adenoma; nonneoplastic nodules (goiter nodule); healthy control	HRMAS NMR	Discrimination of thyroid neoplasia (TC, FA, and NN) from healthy tissues (HC) and benign (FA, NN) from malignant (TC) neoplasia was reported Potential biomarkers/discriminators: (i) TC > FA, NN: phenylalanine, taurine, and lactate; (ii) TC < FA, NN: lipids, choline, phosphocholine, and scyllo- and myo-inositol; (iii) HC > TC (PTC): lipids; (iv) HC < TC (PTC): lactate, alanine

Guo et al., 2014 [58]	Thyroid tissue serum	Thyroid carcinoma (PTC, FTC); benign thyroid tumour (thyroid adenoma and multinodular goiter); healthy control	MALDI-FTICR-IMS MALDI-FTICR-MS	Discrimination between malignant (TC) and being (BTT) tumors, as well as healthy control (HC) was reported Potential biomarkers/discriminators (in tissue and sera): (i) TC, BTT versus HC: PC (34:1); (ii) TC versus BTT: PA (36:3), SM (34:1); (iii) TC versus BTT versus HC: PC (34:1), PA (36:3), and SM (34:1)

Ishikawa et al., 2012 [67]	Thyroid tissue	Thyroid carcinoma (PTC); healthy control	MALDI-IMS	Discrimination between papillary thyroid carcinoma (PTC) and normal tissue (HC) Potential biomarkers/discriminators: (i) PTC > HC: phosphatidylcholine PC (34:1), PC (34:2), and sphingomyelin SM (34:1)

Yao et al., 2011 [118]	Serum	Thyroid carcinoma (PTC); nonneoplastic nodules (goiter nodule); healthy control	LC/MS	Discrimination between thyroid lesions (PTC, NN) and healthy control (HC) Potential biomarkers/discriminators: (i) PTC > NN, HC: 3-hydroxybutyric acid; (ii) PTC > NN: 3-hydroxybutyric acid, FFA (free fatty acids), and acetylcarnitine

TC: thyroid carcinoma; PTC: papillary thyroid carcinoma; FVPTC: follicular variant of papillary thyroid carcinoma; FTC: follicular thyroid carcinoma; FA: follicular adenoma; HC: healthy control; NN: nonneoplastic nodules; BTT: benign thyroid tumor.

## References

[1] Faquin W. C. (2008). The thyroid gland: recurring problems in histologic and cytologic evaluation. *Archives of Pathology and Laboratory Medicine*.

[2] Xing M., Haugen B. R., Schlumberger M. (2013). Progress in molecular-based management of differentiated thyroid cancer. *The Lancet*.

[3] Koperek O., Kornauth C., Capper D. (2012). Immunohistochemical detection of the BRAF V600E-mutated protein in papillary thyroid carcinoma. *The American Journal of Surgical Pathology*.

[4] Zagzag J., Pollack A., Dultz L. (2013). Clinical utility of immunohistochemistry for the detection of the BRAF v600e mutation in papillary thyroid carcinoma. *Surgery*.

[5] Greco A., Borrello M. G., Miranda C., Degl'Innocenti D., Pieroiti M. A. (2009). Molecular pathology of differentiated thyroid cancer. *Quarterly Journal of Nuclear Medicine and Molecular Imaging*.

[6] Weckwerth W. (2003). Metabolomics in systems biology. *Annual Review of Plant Biology*.

[7] Beger R. D. (2013). A review of applications of metabolomics in cancer. *Metabolites*.

[8] Griffin J. L., Shockcor J. P. (2004). Metabolic profiles of cancer cells. *Nature Reviews Cancer*.

[9] Spratlin J. L., Serkova N. J., Eckhardt S. G. (2009). Clinical applications of metabolomics in oncology: a review. *Clinical Cancer Research*.

[10] Howe F. A., Barton S. J., Cudlip S. A. (2003). Metabolic profiles of human brain tumors using quantitative in vivo 1H magnetic resonance spectroscopy. *Magnetic Resonance in Medicine*.

[11] Denkert C., Bucher E., Hilvo M. (2012). Metabolomics of human breast cancer: new approaches for tumor typing and biomarker discovery. *Genome Medicine*.

[12] Kind T., Tolstikov V., Fiehn O., Weiss R. H. (2007). A comprehensive urinary metabolomic approach for identifying kidney cancerr. *Analytical Biochemistry*.

[13] Thysell E., Surowiec I., Hörnberg E. (2010). Metabolomic characterization of human prostate cancer bone metastases reveals increased levels of cholesterol. *PLoS ONE*.

[14] Welker M. J., Orlov D. (2003). Thyroid nodules. *American Family Physician*.

[15] Cibas E. S., Ali S. Z. (2009). The Bethesda system for reporting thyroid cytopathology. *American Journal of Clinical Pathology*.

[16] Mehra P., Verma A. K. (2015). Thyroid cytopathology reporting by the bethesda system: a two-year prospective study in an academic institution. *Pathology Research International*.

[17] Kim T., Oh Y. L., Kim K. M., Shin J. H. (2011). Diagnostic dilemmas of hyalinizing trabecular tumours on fine needle aspiration cytology: a study of seven cases with BRAF mutation analysis. *Cytopathology*.

[18] Bakuła-Zalewska E., Cameron R., Gałczyński J. P., Domanski H. A. (2014). Hyaline matrix in hyalinizing trabecular tumor: findings in fine-needle aspiration smears. *Diagnostic Cytopathology*.

[19] Ustun B., Chhieng D., Prasad M. L. (2014). Follicular variant of papillary thyroid carcinoma: accuracy of FNA diagnosis and implications for patient management. *Endocrine Pathology*.

[20] Jin L., Sebo T. J., Nakamura N. (2006). BRAF mutation analysis in fine needle aspiration (FNA) cytology of the thyroid. *Diagnostic Molecular Pathology*.

[21] Johnson S. J., Hardy S. A., Roberts C., Bourn D., Mallick U., Perros P. (2014). Pilot of *BRAF* mutation analysis in indeterminate, suspicious and malignant thyroid FNA cytology. *Cytopathology*.

[22] Hodak S. P., Rosenthal D. S. (2013). Information for clinicians: commercially available molecular diagnosis testing in the evaluation of thyroid nodule fine-needle aspiration specimens. *Thyroid*.

[23] Krane J. F. (2014). Lessons from early clinical experience with the Afirma gene expression classifier. *Cancer Cytopathology*.

[24] Papotti M., Rodriguez J., de Pompa R., Bartolazzi A., Rosai J. (2005). Galectin-3 and HBME-1 expression in well-differentiated thyroid tumors with follicular architecture of uncertain malignant potential. *Modern Pathology*.

[25] Griffith O. L., Chiu C. G., Gown A. M., Jones S. J. M., Wiseman S. M. (2008). Biomarker panel diagnosis of thyroid cancer: a critical review. *Expert Review of Anticancer Therapy*.

[26] El Demellawy D., Nasr A. L., Babay S., Alowami S. (2009). Diagnostic utility of CD56 immunohistochemistry in papillary carcinoma of the thyroid. *Pathology—Research and Practice*.

[27] Mokhtari M., Eftekhari M., Tahririan R. (2013). Absent CD56 expression in papillary thyroid carcinoma: a finding of potential diagnostic value in problematic cases of thyroid pathology. *Journal of Research in Medical Sciences*.

[28] Shahebrahimi K., Madani S. H., Fazaeli A., Khazaei S., Kanani M., Keshavarz A. (2013). Diagnostic value of CD56 and nm23 markers in papillary thyroid carcinoma. *Indian Journal of Pathology & Microbiology*.

[29] Hedinger C., Williams E. D., Sobin L. H. (1988). Histological typing of thyroid tumors. *International Histological Classification of Tumours*.

[30] Wood S. L., Westbrook J. A., Brown J. E. (2014). Omic-profiling in breast cancer metastasis to bone: Implications for mechanisms, biomarkers and treatment. *Cancer Treatment Reviews*.

[31] Perou C. M., Sørile T., Eisen M. B. (2000). Molecular portraits of human breast tumours. *Nature*.

[32] MacKay A., Weigelt B., Grigoriadis A. (2011). Microarray-based class discovery for molecular classification of breast cancer: analysis of interobserver agreement. *Journal of the National Cancer Institute*.

[33] Su Y., Zheng Y., Zheng W. (2011). Distinct distribution and prognostic significance of molecular subtypes of breast cancer in Chinese women: a population-based cohort study. *BMC Cancer*.

[34] Kao K.-J., Chang K.-M., Hsu H.-C., Huang A. T. (2011). Correlation of microarray-based breast cancer molecular subtypes and clinical outcomes: implications for treatment optimization. *BMC Cancer*.

[35] Huang Y., Prasad M., Lemon W. J. (2001). Gene expression in papillary thyroid carcinoma reveals highly consistent profiles. *Proceedings of the National Academy of Sciences of the United States of America*.

[36] Giordano T. J., Kuick R., Thomas D. G. (2005). Molecular classification of papillary thyroid carcinoma: distinct *BRAF*, *RAS*, and *RET*/*PTC* mutation-specific gene expression profiles discovered by DNA microarray analysis. *Oncogene*.

[37] Aldred M. A., Huang Y., Liyanarachchi S. (2004). Papillary and follicular thyroid carcinomas show distinctly different microarray expression profiles and can be distinguished by a minimum of five genes. *Journal of Clinical Oncology*.

[38] Jarza̧b B., Wiench M., Fujarewicz K. (2005). Gene expression profile of papillary thyroid cancer: sources of variability and diagnostic implications. *Cancer Research*.

[39] Fujarewicz K., Jarza̧b M., Eszlinger M. (2007). A multi-gene approach to differentiate papillary thyroid carcinoma from benign lesions: gene selection using support vector machines with bootstrapping. *Endocrine-Related Cancer*.

[40] Eszlinger M., Krohn K., Kukulska A., Jarza̧b B., Paschke R. (2007). Perspectives and limitations of microarray-based gene expression profiling of thyroid tumors. *Endocrine Reviews*.

[41] Hood B. L., Stewart N. A. S., Conrads T. P. (2009). Development of high-throughput mass spectrometry-based approaches for cancer biomarker discovery and implementation. *Clinics in Laboratory Medicine*.

[42] Fredolini C., Liotta L. A., Petricoin E. F. (2010). Application of proteomic technologies for prostate cancer detection, prognosis, and tailored therapy. *Critical Reviews in Clinical Laboratory Sciences*.

[43] de Wit M., Fijneman R. J. A., Verheul H. M. W., Meijer G. A., Jimenez C. R. (2013). Proteomics in colorectal cancer translational research: biomarker discovery for clinical applications. *Clinical Biochemistry*.

[44] Álvarez-Chaver P., Otero-Estévez O., de la Cadena M. P., Rodríguez-Berrocal F. J., Martínez-Zorzano V. S. (2014). Proteomics for discovery of candidate colorectal cancer biomarkers. *World Journal of Gastroenterology*.

[45] Schneider S. S., Aslebagh R., Wetie A. G., Sturgeon S. R., Darie C. C., Arcaro K. F. (2014). Using breast milk to assess breast cancer risk: the role of mass spectrometry-based proteomics. *Advances in Experimental Medicine and Biology*.

[46] Krause K., Jeßnitzer B., Fuhrer D. (2009). Proteomics in thyroid tumor research. *Journal of Clinical Endocrinology and Metabolism*.

[47] Brown L. M., Helmke S. M., Hunsucker S. W. (2006). Quantitative and qualitative differences in protein expression between papillary thyroid carcinoma and normal thyroid tissue. *Molecular Carcinogenesis*.

[48] Netea-Maier R. T., Hunsucker S. W., Hoevenaars B. M. (2008). Discovery and validation of protein abundance differences between follicular thyroid neoplasms. *Cancer Research*.

[49] Hanahan D., Weinberg R. A. (2011). Hallmarks of cancer: the next generation. *Cell*.

[50] Weljie A. M., Jirik F. R. (2011). Hypoxia-induced metabolic shifts in cancer cells: moving beyond the Warburg effect. *International Journal of Biochemistry and Cell Biology*.

[51] Heiden M. G. V., Cantley L. C., Thompson C. B. (2009). Understanding the warburg effect: the metabolic requirements of cell proliferation. *Science*.

[52] Warburg O. (1956). On the origin of cancer cells. *Science*.

[53] Sitter B., Sonnewald U., Spraul M., Fjösne H. E., Gribbestad I. S. (2002). High-resolution magic angle spinning MRS of breast cancer tissue. *NMR in Biomedicine*.

[54] Swanson M. G., Zektzer A. S., Tabatabai Z. L. (2006). Quantitative analysis of prostate metabolites using ^1^H HR-MAS spectroscopy. *Magnetic Resonance in Medicine*.

[55] Miccoli P., Torregrossa L., Shintu L. (2012). Metabolomics approach to thyroid nodules: a high-resolution magic-angle spinning nuclear magnetic resonance-based study. *Surgery*.

[56] Menendez J. A., Lupu R. (2007). Fatty acid synthase and the lipogenic phenotype in cancer pathogenesis. *Nature Reviews Cancer*.

[57] Hilvo M., Denkert C., Lehtinen L. (2011). Novel theranostic opportunities offered by characterization of altered membrane lipid metabolism in breast cancer progression. *Cancer Research*.

[58] Guo S., Qiu L., Wang Y. (2014). Tissue imaging and serum lipidomic profiling for screening potential biomarkers of thyroid tumors by matrix-assisted laser desorption/ionization-Fourier transform ion cyclotron resonance mass spectrometry. *Analytical and Bioanalytical Chemistry*.

[59] Cairns R. A., Harris I. S., Mak T. W. (2011). Regulation of cancer cell metabolism. *Nature Reviews Cancer*.

[60] Metallo C. M., Gameiro P. A., Bell E. L. (2012). Reductive glutamine metabolism by IDH1 mediates lipogenesis under hypoxia. *Nature*.

[61] Glunde K., Bhujwalla Z. M., Ronen S. M. (2011). Choline metabolism in malignant transformation. *Nature Reviews Cancer*.

[62] Bishop W. R., Bell R. M. (1988). Assembly of phospholipids into cellular membranes: biosynthesis, transmembrane movement and intracellular translocation. *Annual Review of Cell Biology*.

[63] Dória M. L., Cotrim Z., MacEdo B. (2012). Lipidomic approach to identify patterns in phospholipid profiles and define class differences in mammary epithelial and breast cancer cells. *Breast Cancer Research and Treatment*.

[64] Glunde K., Jie C., Bhujwalla Z. M. (2004). Molecular causes of the aberrant choline phospholipid metabolism in breast cancer. *Cancer Research*.

[65] Ackerstaff E., Pflug B. R., Nelson J. B., Bhujwalla Z. M. (2001). Detection of increased choline compounds with proton nuclear magnetic resonance spectroscopy subsequent to malignant transformation of human prostatic epithelial cells. *Cancer Research*.

[66] Herminghaus S., Pilatus U., Möller-Hartmann W. (2002). Increased choline levels coincide with enhanced proliferative activity of human neuroepithelial brain tumors. *NMR in Biomedicine*.

[67] Ishikawa S., Tateya I., Hayasaka T. (2012). Increased expression of phosphatidylcholine (16:0/18:1) and (16:0/18:2) in thyroid papillary cancer. *PLoS ONE*.

[68] Kuesel A. C., Donnelly S. M., Halliday W., Sutherland G. R., Smith I. C. P. (1994). Mobile lipids and metabolic heterogeneity of brain tumours as detectable by ex vivo 1H MR spectroscopy. *NMR in Biomedicine*.

[69] Fernandis A. Z., Wenk M. R. (2009). Lipid-based biomarkers for cancer. *Journal of Chromatography B: Analytical Technologies in the Biomedical and Life Sciences*.

[70] Jelonek K., Ros M., Pietrowska M., Widlak P. (2013). Cancer biomarkers and mass spectrometry-based analyses of phospholipids in body fluids. *Clinical Lipidology*.

[71] Brizel D. M., Schroeder T., Scher R. L. (2001). Elevated tumor lactate concentrations predict for an increased risk of metastases in head-and-neck cancer. *International Journal of Radiation Oncology, Biology, Physics*.

[72] Walenta S., Schroeder T., Mueller-Klieser W. (2004). Lactate in solid malignant tumors: potential basis of a metabolic classification in clinical oncology. *Current Medicinal Chemistry*.

[73] Tessem M.-B., Swanson M. G., Keshari K. R. (2008). Evaluation of lactate and alanine as metabolic biomarkers of prostate cancer using 1H HR-MAS spectroscopy of biopsy tissues. *Magnetic Resonance in Medicine*.

[74] Gribbestad I. S., Sitter B., Lundgren S., Krane J., Axelson D. (1999). Metabolite composition in breast tumors examined by proton nuclear magnetic resonance spectroscopy. *Anticancer Research*.

[75] Sitter B., Lundgren S., Bathen T. F., Halgunset J., Fjosne H. E., Gribbestad I. S. (2006). Comparison of HR MAS MR spectroscopic profiles of breast cancer tissue with clinical parameters. *NMR in Biomedicine*.

[76] Bartella L., Thakur S. B., Morris E. A. (2007). Enhancing nonmass lesions in the breast: evaluation with proton (^1^H) MR spectroscopy. *Radiology*.

[77] Bathen T. F., Jensen L. R., Sitter B. (2007). MR-determined metabolic phenotype of breast cancer in prediction of lymphatic spread, grade, and hormone status. *Breast Cancer Research and Treatment*.

[78] Florian C. L., Preece N. E., Bhakoo K. K., Williams S. R., Noble M. D. (1995). Cell type-specific fingerprinting of meningioma and meningeal cells by proton nuclear magnetic resonance spectroscopy. *Cancer Research*.

[79] Maxwell R. J., Martínez-Pérez I., Cerdán S. (1998). Pattern recognition analysis of 1H NMR spectra from perchloric acid extracts of human brain tumor biopsies. *Magnetic Resonance in Medicine*.

[80] Dowling C., Bollen A. W., Noworolski S. M. (2001). Preoperative proton MR spectroscopic imaging of brain tumors: correlation with histopathologic analysis of resection specimens. *American Journal of Neuroradiology*.

[81] Cheng L. L., Wu C.-L., Smith M. R., Gonzalez R. G. (2001). Non-destructive quantitation of spermine in human prostate tissue samples using HRMAS 1H NMR spectroscopy at 9.4 T. *FEBS Letters*.

[82] Dunn W. B., Bailey N. J. C., Johnson H. E. (2005). Measuring the metabolome: current analytical technologies. *Analyst*.

[83] Fiehn O., Robertson D., Griffin J. (2007). The metabolomics standards initiative (MSI). *Metabolomics*.

[84] Griffin J. L., Nicholls A. W., Daykin C. A. (2007). Standard reporting requirements for biological samples in metabolomics experiments: Mammalian/in vivo experiments. *Metabolomics*.

[85] Sumner L. W., Amberg A., Barrett D. (2007). Proposed minimum reporting standards for chemical analysis. *Metabolomics*.

[86] Rubtsov D. V., Jenkins H., Ludwig C. (2007). Proposed reporting requirements for the description of NMR-based metabolomics experiments. *Metabolomics*.

[87] Goodacre R., Broadhurst D., Smilde A. K. (2007). Proposed minimum reporting standards for data analysis in metabolomics. *Metabolomics*.

[88] Wu H., Xue R., Tang Z. (2010). Metabolomic investigation of gastric cancer tissue using gas chromatography/mass spectrometry. *Analytical and Bioanalytical Chemistry*.

[89] Seeley E. H., Caprioli R. M. (2011). MALDI imaging mass spectrometry of human tissue: method challenges and clinical perspectives. *Trends in Biotechnology*.

[90] Rocha C. M., Barros A. S., Gil A. M. (2010). Metabolic profiling of human lung cancer tissue by ^1^H high resolution magic angle spinning (HRMAS) NMR spectroscopy. *Journal of Proteome Research*.

[91] Wu H., Southam A. D., Hines A., Viant M. R. (2008). High-throughput tissue extraction protocol for NMR- and MS-based metabolomics. *Analytical Biochemistry*.

[92] Cheng L., Zhang S., MacLennan G. T. (2013). Laser-assisted microdissection in translational research: theory, technical considerations, and future applications. *Applied Immunohistochemistry and Molecular Morphology*.

[93] Kelly A. D., Breitkopf S. B., Yuan M., Goldsmith J., Spentzos D., Asara J. M. (2011). Metabolomic profiling from formalin-fixed, paraffin-embedded tumor tissue using targeted LC/MS/MS: application in sarcoma. *PLoS ONE*.

[94] Dettmer K., Aronov P. A., Hammock B. D. (2007). Mass spectrometry-based metabolomics. *Mass Spectrometry Reviews*.

[95] Jiye A., Trygg J., Gullberg J. (2005). Extraction and GC/MS analysis of the human blood plasma metabolome. *Analytical Chemistry*.

[96] Serkova N. J., Spratlin J. L., Eckhardt S. G. (2007). NMR-based metabolomics: translational application and treatment of cancer. *Current Opinion in Molecular Therapeutics*.

[97] Villas-Bôas S. G., Mas S., Åkesson M., Smedsgaard J., Nielsen J. (2005). Mass spectrometry in metabolome analysis. *Mass Spectrometry Reviews*.

[98] Stobiecki M. (2000). Application of mass spectrometry for identification and structural studies of flavonoid glycosides. *Phytochemistry*.

[99] Kouremenos K. A., Johansson M., Marriott P. J. (2012). Advances in gas chromatographic methods for the identification of biomarkers in cancer. *Journal of Cancer*.

[100] Chan E. C. Y., Koh P. K., Mal M. (2009). Metabolic profiling of human colorectal cancer using high-resolution magic angle spinning nuclear magnetic resonance (HR-MAS NMR) spectroscopy and gas chromatography mass spectrometry (GC/MS). *Journal of Proteome Research*.

[101] Glunde K., Bhujwalla Z. M. (2011). Metabolic tumor imaging using magnetic resonance spectroscopy. *Seminars in Oncology*.

[102] Lietz C. B., Gemperline E., Li L. (2013). Qualitative and quantitative mass spectrometry imaging of drugs and metabolites. *Advanced Drug Delivery Reviews*.

[103] Ellis S. R., Brown S. H., Panhuis M. I. H., Blanksby S. J., Mitchell T. W. (2013). Surface analysis of lipids by mass spectrometry: more than just imaging. *Progress in Lipid Research*.

[104] Sun N., Walch A. (2013). Qualitative and quantitative mass spectrometry imaging of drugs and metabolites in tissue at therapeutic levels. *Histochemistry and Cell Biology*.

[105] Katajamaa M., Orešič M. (2007). Data processing for mass spectrometry-based metabolomics. *Journal of Chromatography A*.

[106] Ellinger J. J., Chylla R. A., Ulrich E. L., Markley J. L. (2013). Databases and software for NMR-based metabolomics. *Current Metabolomics*.

[107] Wishart D. S., Tzur D., Knox C. (2007). HMDB: The Human Metabolome Database. *Nucleic Acids Research*.

[108] Smith C. A., O'Maille G., Want E. J. (2005). METLIN: A metabolite mass spectral database. *Therapeutic Drug Monitoring*.

[109] Kopka J., Schauer N., Krueger S. (2005). GMD@CSB.DB: The Golm Metabolome Database. *Bioinformatics*.

[110] Horai H., Arita M., Kanaya S. (2010). MassBank: a public repository for sharing mass spectral data for life sciences. *Journal of Mass Spectrometry*.

[111] Fahy E., Sud M., Cotter D., Subramaniam S. (2007). LIPID MAPS online tools for lipid research. *Nucleic Acids Research*.

[112] Lindon J. C., Holmes E., Nicholson J. K. (2001). Pattern recognition methods and applications in biomedical magnetic resonance. *Progress in Nuclear Magnetic Resonance Spectroscopy*.

[113] Madsen R., Lundstedt T., Trygg J. (2010). Chemometrics in metabolomics—a review in human disease diagnosis. *Analytica Chimica Acta*.

[114] Blekherman G., Laubenbacher R., Cortes D. F. (2011). Bioinformatics tools for cancer metabolomics. *Metabolomics*.

[115] Carpi A., Mechanick J. I., Saussez S., Nicolini A. (2010). Thyroid tumor marker genomics and proteomics: diagnostic and clinical implications. *Journal of Cellular Physiology*.

[116] Deja S., Dawiskiba T., Balcerzak W. (2013). Follicular adenomas exhibit a unique metabolic profile. ^1^H NMR studies of thyroid lesions. *PLoS ONE*.

[117] Torregrossa L., Shintu L., Nambiath Chandran J. (2012). Toward the reliable diagnosis of indeterminate thyroid lesions: a HRMAS NMR-based metabolomics case of study. *Journal of Proteome Research*.

[118] Yao Z., Yin P., Su D. (2011). Serum metabolic profiling and features of papillary thyroid carcinoma and nodular goiter. *Molecular BioSystems*.

[119] Russell P., Lean C. L., Delbridge L., May G. L., Dowd S., Mountford C. E. (1994). Proton magnetic resonance and human thyroid neoplasia I: discrimination between benign and malignant neoplasms. *The American Journal of Medicine*.

[120] Delbridge L., Lean C. L., Russell P. (1994). Proton magnetic resonance and human thyroid neoplasia II: potential avoidance of surgery for benign follicular neoplasms. *World Journal of Surgery*.

[121] Lean C. L., Delbridge L., Russell P. (1995). Diagnosis of follicular thyroid lesions by proton magnetic resonance on fine needle biopsy. *The Journal of Clinical Endocrinology & Metabolism*.

[122] Mackinnon W. B., Delbridge L., Russell P. (1996). Two-dimensional proton magnetic resonance spectroscopy for tissue characterization of thyroid neoplasms. *World Journal of Surgery*.

[123] Raffelt K., Moka D., Süllentrop F., Dietlein M., Hahn J., Schicha H. (2000). Systemic alterations in phospholipid concentrations of blood plasma in patients with thyroid carcinoma: an *in-vitro*
^31^P high-resolution NMR study. *NMR in Biomedicine*.

[124] Yoshioka Y., Sasaki J., Yamamoto M., Saitoh K., Nakaya S., Kubokawa M. (2000). Quantitation by ^1^H-NMR of dolichol, cholesterol and choline-containing lipids in extracts of normal and phathological thyroid tissue. *NMR in Biomedicine*.

[125] Jordan K. W., Adkins C. B., Cheng L. L., Faquin W. C. (2011). Application of magnetic-resonance-spectroscopy-based metabolomics to the fine-needle aspiration diagnosis of papillary thyroid carcinoma. *Acta Cytologica*.

[126] Elshafey R., Elattar A., Mlees M., Esheba N. (2014). Role of quantitative diffusion-weighted MRI and ^1^H MR spectroscopy in distinguishing between benign and malignant thyroid nodules. *The Egyptian Journal of Radiology and Nuclear Medicine*.

[127] Guo Y., Wang X., Qiu L. (2012). Probing gender-specific lipid metabolites and diagnostic biomarkers for lung cancer using Fourier transform ion cyclotron resonance mass spectrometry. *Clinica Chimica Acta*.

[128] Li F., Qin X., Chen H. (2013). Lipid profiling for early diagnosis and progression of colorectal cancer using direct-infusion electrospray ionization Fourier transform ion cyclotron resonance mass spectrometry. *Rapid Communications in Mass Spectrometry*.

[129] King A. D., Yeung D. K. W., Ahuja A. T. (2005). In vivo ^1^H MR spectroscopy of thyroid carcinoma. *European Journal of Radiology*.

[130] Gupta N., Kakar A. K., Chowdhury V., Gulati P., Shankar L. R., Vindal A. (2007). Magnetic resonance spectroscopy as a diagnostic modality for carcinoma thyroid. *European Journal of Radiology*.

[131] Ide Y., Waki M., Hayasaka T. (2013). Human Breast cancer tissues contain abundant phosphatidylcholine(36:1) with high stearoyl-CoA desaturase-1 expression. *PLoS ONE*.

[132] von Roemeling C. A., Marlow L. A., Wei J. J. (2013). Stearoyl-CoA desaturase 1 is a novel molecular therapeutic target for clear cell renal cell carcinoma. *Clinical Cancer Research*.

[133] Gupta M. K., Singh D. B., Rath S. K., Misra K. (2012). Metabolic modeling and simulation analysis of thyroid disorder pathway. *Journal of Computer Science & Systems Biology*.

[134] Gupta M. K., Singh D. B., Shukla R., Misra K. (2013). A comprehensive metabolic modeling of thyroid pathway in relation to thyroid pathophysiology and therapeutics. *OMICS: A Journal of Integrative Biology*.

